# Supplementary material for: Partnering With Interpreter Services: Standardized Patient Cases to Improve Communication With Limited English Proficiency Patients
Source: MedEdPORTAL. 2019 May 20;15:10826. doi: 10.15766/mep_2374-8265.10826 (PMC6543860; doi:10.15766/mep_2374-8265.10826)
Supplement: Supplementary file 1 — A. Case 1 SP Information.docx B. Case 2 SP Information.docx C. Case 1 Resident Participant Information.docx D. Case 2 Resident Participant Information.docx E. Case 1 Physical Exam Sheet.docx F. Case 2 Physical Exam Sheet.docx G. UCI Interpreter Scale.docx H. UCI Interpreter Impact Rating Scale.docx I. Resident Session Evaluation Form.docx J. OSCE Workshop Schedule.docx K. UCI FORS Scale.docx L. Case 1 Observer Checklist.xlsx M. Case 2 Observer Checklist.xlsx [file mep-15-10826-s001.zip › _Educational Summary Report_10826.pdf]

MedEdPORTAL®
The Journal of Teaching and Learning Resources

Original Publication

OPEN ACCESS

Partnering With Interpreter Services: Standardized Patient Cases to Improve Communication With Limited English Proficiency Patients

Emily Pinto Taylor, MD*, Arielle Mulenos, Avik Chatterjee, MD, MPH, Jaideep S. Talwalkar, MD
*Corresponding author: emily.pintotaylor@yale.edu

Abstract

Introduction: As the number of patients with limited English proficiency grows, there is increasing awareness in the medical community about disparities in health outcomes for this population. The proper use of professional medical interpreters improves communication between physicians and patients with limited English proficiency. Typically, however, little curricular time in medical training is devoted to this competency. **Methods:** We developed a two-station objective structured clinical examination (OSCE) in which learners worked with interpreters to conduct medical interviews with Spanish-speaking standardized patients (SPs). Cases were designed for use with residents from any medical specialty and to have personal and emotional richness in keeping with the real-life circumstances of many patients. Twelve residents from six medical specialties completed a session evaluation and were assessed by faculty, SPs, and interpreters using existing validated instruments and case-specific checklists. **Results:** All residents reported that the cases mimicked real patient encounters. The checklists were well received and easy to use. While scores varied between residents, deficiencies were identified in basic communication skills for interacting with a non-English-speaking SP through an interpreter, including maintaining proper eye contact and open body posture with patients and introducing and clearly articulating the role of the interpreter. **Discussion:** A two-station OSCE utilizing professional medical interpreters and Spanish-speaking SPs was well received by all participants. Residents' scores helped identify common skill gaps in their work with interpreters. Based on the success of the pilot deployment, we plan to target educational interventions at these common deficiencies and offer the OSCE to more trainees.

Keywords

OSCE, Standardized Patient, Limited English Proficiency, Spanish, Interpreter Use, Cultural Competence, Health Equity, Interpreter Communication

Educational Objectives

By the end of this activity, learners will be able to:

1. Define the role of the interpreter in a clinical encounter.
2. Utilize an interpreter to obtain a history from a Spanish-speaking standardized patient.
3. Demonstrate best practices while working with an interpreter, including using second-person language, speaking in short phrases, showing appropriate body language, and minimizing medical jargon.

Introduction

Effective communication between physicians and patients is essential to the provision of high-quality, patient-centered health care.[1,2] Language barriers encountered in the care of individuals with limited English proficiency (LEP), a growing segment of the US population, adversely affect health care quality.[3-5] According to the US Census, in 2011, there were 25.3 million LEP individuals, both foreign born and US born, residing in the country, and the LEP share of the population has grown from six percent in 1990 to nine percent in 2010, which primarily reflects Spanish speakers.[6] Despite growing awareness of worse health outcomes for LEP patients, increasing numbers of non-English-speaking patients seen by clinicians

Citation: Pinto Taylor E, Mulenos A, Chatterjee A, Talwalkar JS. Partnering with interpreter services: standardized patient cases to improve communication with limited English proficiency patients. *MedEdPORTAL*. 2019;15:10826. https://doi.org/10.15766/mep_2374-8265.10826

Copyright: © 2019 Pinto Taylor et al. This is an open-access article distributed under the terms of the Creative Commons Attribution license.

Appendices

A. Case 1 SP Information.docx
B. Case 2 SP Information.docx
C. Case 1 Resident Participant Information.docx
D. Case 2 Resident Participant Information.docx
E. Case 1 Physical Exam Sheet .docx
F. Case 2 Physical Exam Sheet.docx
G. UCI Interpreter Scale.docx
H. UCI Interpreter Impact Rating Scale.docx
I. Resident Session Evaluation Form.docx
J. OSCE Workshop Schedule .docx
K. UCI FORS Scale.docx
L. Case 1 Observer Checklist .xlsx
M. Case 2 Observer Checklist .xlsx

All appendices are peer reviewed as integral parts of the Original Publication.

AAMC

in the health care setting, and recognition of the importance of culturally competent care, there is typically little curricular time in medical training devoted to the effective use of interpreters.[7]

Standardized patients (SPs) are routinely utilized in medical education to present learners with opportunities to develop, practice, and demonstrate skills in a contrived setting that simulates the real clinical environment.[8] Objective structured clinical examinations (OSCEs) utilizing SPs allow for assessment of learners at high levels on Miller's pyramid; learners can show and do rather than simply demonstrate knowledge.[9] There are only a few *MedEdPORTAL* publications of SP cases designed for use by Spanish-speaking actors,[10,11] and the proportion compared to cases for English-speaking actors does not reflect the rising share of the population occupied by Spanish-speaking individuals. These existing publications focus on verification of fluency in undergraduate medical learners (without certified medical interpreter involvement)[10] or illustrate the difference between using an in-person or telephone interpreter, compared with a bilingual family member.[11] Existing resources are not designed to assess residents' abilities to use a certified medical interpreter or to illustrate best practices for doing so through evaluation on multiple metrics. The incorporation of interpreters into the SP encounter allows for a more accurate simulation of a real patient visit, and improving residents' skills with interpreters is an important step in global improvement of physician-patient communication.[12]

To address the need for incorporation of interpreters into SP encounters with residents, we developed two cases (Appendices A and B) targeted at postgraduate resident learners, in which Spanish native-speaking actors served as SPs and certified medical interpreters from our academic medical center served in their typical capacity as interpreters. The cases were initially designed as an assessment mechanism as part of our yearlong, resident-led Spanish language curriculum, which is described below.[13] The purpose of the current assessment was to demonstrate feasibility of the two cases on a small scale with residents from multiple specialties, irrespective of their participation status in the Spanish language curriculum.

Methods

Curricular Context

We designed two cases for an OSCE as part of the broader Yale Resident Spanish Initiative (YRSI). The YRSI is a yearlong, self-directed Spanish language curriculum for residents designed to improve language skills and understanding of health-related cultural beliefs.[13,14] Our residents generally begin the program with some Spanish language skills and desire to grow in their proficiency throughout the year, through our online self-paced modules, supplementary video materials, and language tutoring in person with native Spanish speakers. One unintended outcome of YRSI is that hospital interpreters have observed that residents participating in YRSI appear more comfortable utilizing interpreters during patient encounters than residents who do not participate in YRSI.[15] Therefore, we initially created the OSCE to assess the impact of YRSI on residents' use of medical interpreters by measuring their effectiveness of interpreter use during an SP interview. The OSCE in this pilot project was administered at the start of the curriculum (October 2017) and 6 months later (April 2018). Participants represented a mix of residents enrolled and not enrolled in YRSI. None had received specific training related to use of medical interpreters as part of YRSI prior to the OSCE or during the study period.

Case Development

We conceived of the two cases, which then went through many rounds of revision with the SP program team and eventually with the SPs themselves. Choosing topics sufficiently basic to be addressed by residents from any specialty, we designed the cases to be portrayed by either a man or woman and to have personal and emotional richness in keeping with the real-life circumstances of many patients seen in our emergency department and urgent care clinics. The final cases were patients presenting to an urgent care setting with abdominal pain (Appendix A) and back pain (Appendix B).

After case development, the full project was submitted to the institutional review board for approval and granted a status of exempt, allowing us to proceed with SP training and participant recruitment.

Actor and Interpreter Recruitment and Training

We recruited four native Spanish speakers as SPs to most accurately reflect the barrier in physician-patient communication seen with LEP patients. Actors were offered the standard rate of payment as part of the SP program, though some actors chose to volunteer their time. SPs received their cases in advance and were told to review the script prior to a 2-hour training session with the director of the SP program at our institution.

The training session first focused on the details of the cases, including questions and suggestions brought up by the SPs. SPs provided helpful feedback to add to the authenticity of the cases based on their lived experience, noting nuances in work status with certain types of visas and emotional responses concerning immigration status. We then reviewed session logistics. During the remainder of the training, the SPs were coached to do the following:

- Speak only in Spanish during the OSCE and reveal no English comprehension (despite their fluency in English as a second language).
- Defer all responses until after the interpreter had completed the interpretation of the physician's statements.
- Reveal potentially sensitive information (e.g., immigration status, employment, family life) only if asked or if the physician created an environment in which such disclosures would feel natural and safe.
- Use the end-of-session assessment checklists.

We recruited interpreters from the language services department at our hospital and paid them an hourly rate similar to that offered to the SPs. Since they were being asked to interpret as they would with any real patient encounter, we provided no specific training other than a 10-minute overview of logistics prior to the OSCE. The interpreters were encouraged to act just as they would for a typical patient encounter and to stand or sit where they felt most comfortable.

The cost of recruiting, training, and hiring the actors and interpreters was approximately $1,200, covering 2 hours of training for SPs and 2 hours of OSCEs for four SPs and four interpreters, repeated in fall and spring sessions.

Resident Recruitment

We recruited 12 resident physicians from different training programs, postgraduate year levels, and participation statuses in the YRSI. Residents were recruited from internal medicine, pediatrics, general surgery, emergency medicine, urology, and radiation oncology. They were given no specific training related to the OSCE other than a 2-minute overview of logistics immediately before each session and received an $80 honorarium for their participation ($40 for each session).

Session Logistics

The OSCE occurred over two evenings as stand-alone sessions and was run with four of the 12 resident participants at a time. Each evening, residents rotated through the OSCE in three waves, each completing one of the two cases per session. As a result, four rooms were operating simultaneously with a resident, SP, and interpreter in each. Rooms were labeled A, B, C, and D.

We utilized rooms in our university's clinical exam practice suite. The rooms were equipped with audiovisual capability and simulated an outpatient office setting. Before entering the exam room, residents received a file folder labeled A, B, C, or D, corresponding to the room and case for the encounter. The folder contained basic case details and instructions (Appendices C and D). As noted above, prior to the

start of each wave, a member of the OSCE team spent 2 minutes explaining logistics to the residents. The important details communicated at this point included the time allotment of 15 minutes for the case and an explanation of the instructions contained in the resident folders. Specifically, residents learned that they would focus their attention on history and counseling during the OSCE. For the physical examination, residents would simply tell the SP that they would like to perform a physical examination, at which point the SP would hand them a checklist containing the relevant findings (Appendices E and F).

After reviewing the case details outside the room, the resident met the interpreter, and they entered the room together. After 15 minutes, residents were informed that the encounter was finished, at which time the interpreters and SPs were given 5 minutes to complete a rating scale (Appendices G and H) and the residents completed a session evaluation (Appendix I).

At the end of the wave, there was a 5-minute window for transition, during which the previous group of residents exited while the next group arrived. On each evening of the OSCE, each SP and interpreter completed their encounter three times with three different residents during a roughly 90-minute workshop (Appendix J). The OSCE was repeated 6 months after the first session, with residents completing the case they had not completed during the first session. One additional staff member was required to keep the sessions on schedule, review the logistics with participants, and file the video footage upon completion of the OSCE.

Learner Assessment

Following each encounter, interpreters and SPs completed rating scales to evaluate the resident participant and his/her effectiveness in using interpreters and communicating with an LEP patient. Interpreters utilized the University of California, Irvine, School of Medicine (UCISOM) Interpreter Scale (IS, Appendix G),[16] while SPs utilized the Interpreter Impact Rating Scale (IIRS, Appendix H).[17]

The IS is a 13-question validated instrument designed to measure communication skills as observed by trained interpreters, focusing on objective verbal and observable behaviors and minimizing interpreters' emotional responses to questions. The IIRS is a seven-question validated instrument designed for use by SPs in any language encounter; it emphasizes patient-centeredness and observable behaviors as a patient with emotional engagement in the encounter.

Once both OSCE sessions had been completed, the videos of each encounter were reviewed by a faculty member using the Faculty Observer Rating Scale (FORS, Appendix K), an 11-question validated instrument designed for use by faculty not proficient in the language of the encounter, independent of the clinical case, and reflective of objective observation of communication skill behaviors.[16] The faculty member also utilized case-specific checklists (Appendices L and M) designed by ourselves and SP program staff to measure the number of case elements elicited during each interview. The case-specific checklists were designed to be dense, so as to allow for differentiation in scores between participants, because an evaluation of process without accounting for content would provide an incomplete picture of communication competency in a clinical encounter. Finally, the faculty reviewer made unstructured observations to be provided back to the residents as formative feedback.

We utilized and scored the IS, IIRS, and FORS as described by Lie and colleagues in their validation studies.[16,17] These instruments utilized a 5-point Likert scale (1 = *marginal/poor*, 5 = *outstanding*) to gauge performance on each response item. All raters (interpreters, SPs, and faculty) reviewed the checklists in advance and had opportunities to ask clarifying questions. For example, because in practice many interpreters introduce themselves, raters were told to give residents credit for having introduced the interpreter even if the interpreter had initiated the introduction. However, if the interpreter had to interrupt the resident because the resident had not introduced the interpreter, then the resident was not given credit. We report mean and standard deviations for the IS, IIRS, and FORS in the Results section, below.

OSCE Assessment

Residents completed a postsession evaluation after each OSCE—the Resident Session Evaluation Form (Appendix I). We used this resident evaluation form internally in order to correlate residents' perceived ability with their scores on the case-specific checklists and did not run correlations on the self-assessment of residents' perception of their performance with the actual assessment scores. We conducted unstructured interviews of the interpreters and SPs during and after the first OSCE and held a focus group for interpreters and SPs after the second OSCE. We asked interpreters and SPs to comment on their overall impression of the sessions, logistics, and their interest in future participation.

Results

On the Resident Session Evaluation Form, all 12 resident physicians felt the SP encounters were effective or very effective at simulating a real encounter with an LEP patient, including the inherent challenges with time, cultural differences, and role of an interpreter. On a 5-point Likert scale (1 = *not effective*, 5 = *very effective*), residents' mean score of the effectiveness of the OSCE in mimicking a real patient encounter was 4.9, and their mean score of overall experience with the OSCE was 4.8.

During interviews and focus groups, interpreters and SPs, respectively, found the IS and IIRS intuitive and easy to use. All parties felt that the simulated encounter was an important topic for medical trainees, that it was well organized, that the cases were realistic and well written, and that training was adequate. Some participants were surprised that many residents used only a portion of the time available for the encounters, though others noted that the pace of the encounters reflected real life. Multiple SPs and interpreters commented that residents did not address the cultural beliefs and questions of the patients. All SPs and interpreters were enthusiastic about the prospect of getting involved in other curricular offerings related to working with LEP patients.

The faculty reviewer found the FORS and case-specific checklists easy to use. Suggestions for improving the case-specific checklists emerged based on items that came up in some interviews. For example, some residents appropriately inquired about domestic violence, though this was not an item on the case-specific checklist.

Overall, combined mean scores on the IS, IIRS, and FORS ranged from 4.0 to 4.8 on 5-point Likert scales (1 = *marginal/poor,* 5 = *outstanding*; Table 1). Resident performance on the IS and FORS was not significantly different; however, a significant difference was found on the IIRS, with higher scores noted by residents participating in the back pain case. Resident scores on the case-specific checklists varied widely, though all showed room for improvement (Table 1). Item-specific analysis of the UCISOM checklists revealed that the most common competencies where residents struggled were maintaining proper eye contact and open body language with patients and introducing and clearly articulating the role of the interpreter (Table 2, Table 3, and Table 4). Because this OSCE was developed as a part of the broader YRSI curriculum, we conducted a separate analysis of differences in checklist scores based on YRSI participation status, which we have described elsewhere.[18]

Table 1. Mean Checklist Scores of Participants (N = 12)

	Case 1: Abdominal Pain		Case 2: Back Pain		
**Checklist**	***M* (*SD*)[a]**	**% correct (*SD*)**	***M* (*SD*)[a]**	**% correct (*SD*)**	***p***
University of California, Irvine, School of Medicine:					
Interpreter Scale	4.0 (0.7)		4.0 (0.7)		.82
Interpreter Impact Rating Scale	4.4 (0.3)		4.8 (0.1)		.02
Faculty Observer Rating Scale	4.6 (0.4)		4.4 (0.5)		.34
Case-specific checklist of history and counseling items		37.3 (7.0)		40.2 (13.4)	.09

[a]Rated on a 5-point Likert scale (1 = *marginal/poor,* 5 = *outstanding*).

Table 2. Interpreter Scale Item-Specific Scores for Participants (*N* = 12)

Item	*M* (*SD*)[a]	
	**Case 1: Abdominal Pain**	**Case 2: Back Pain**
The trainee:		
Introduced himself or herself to me.	Yes: 8[b]	Yes: 9[b]
Introduced me to the patient.	Yes: 7[b]	Yes: 5[b]
Adequately explained the purpose of the interview.	3.0 (1.4)	3.4 (1.1)
Explained my role to the patient at the beginning.	2.9 (1.4)	2.1 (0.9)
Arranged the seating in a manner conducive to effective interpretation.	3.9 (0.7)	3.7 (1.3)
Asked the patient one question at a time.	4.6 (0.7)	4.3 (1.0)
Listened to me as I interpreted the patient's answers, without unnecessary interruption.	4.9 (0.3)	4.6 (0.7)
Asked questions to clarify his/her own understanding of the patient's answers.	3.5 (1.2)	4.3 (0.7)
Asked the patient if he/she had any questions.	4.4 (1.2)	4.7 (0.5)
Maintained direct eye contact with the patient (instead of me) most of the time.	4.2 (0.4)	4.3 (0.6)
Addressed the patient in the first person and not as "he/she."	5.0 (0)	4.6 (0.5)
Kept me "on track" (i.e., questioned me when lapses led to incomplete interpretations).	4.1 (0.8)	3.7 (0.9)
Rate your OVERALL SATISFACTION with the encounter.	4.1 (0.8)	4.3 (0.9)

[a]Rated on a 5-point Likert scale (1 = *marginal/low*, 5 = *outstanding*).
[b]On yes/no items, descriptions indicate the number of participants out of 12 total who scored yes in lieu of mean and standard deviation.

Table 3. Interpreter Impact Rating Scale Item-Specific Scores for Participants (*N* = 12)

Item	*M* (*SD*)[a]	
	**Case 1: Abdominal Pain**	**Case 2: Back Pain**
The trainee:		
Made direct eye contact with me during the encounter instead of with the interpreter most of the time.	4.3 (0.9)	4.6 (0.7)
Directly addressed the issues translated that were of concern to me.	4.5 (0.5)	4.8 (0.4)
Acknowledged and responded to my beliefs, concerns, and expectations about my problems.	4.3 (0.6)	4.7 (0.5)
Asked me questions in the first person (example: "Do you feel . . ." rather than "Interpreter, can you ask him if he . . .").	4.7 (0.5)	4.9 (0.3)
Sat at a comfortable distance from me (not too close and not too far away).	4.6 (0.5)	4.9 (0.3)
Nonverbal body communication (e.g., mannerisms, facial expressions, body language) was reassuring.	4.1 (1.1)	4.6(0.7)
Rate your OVERALL SATISFACTION with the encounter.	4.1 (1.0)	4.8 (0.5)

[a]Rated on a 5-point Likert scale (1 = *marginal/low*, 5 = *outstanding*).

Table 4. Faculty Observer Rating Scale Item-Specific Scores for Participants (*N* = 12)

Item	*M* (*SD*)[a]	
	**Case 1: Abdominal Pain**	**Case 2: Back Pain**
The trainee:		
Adequately explained the purpose of the interview to the interpreter.	3.5 (0.8)	3.4 (0.7)
Explained the interpreter's role to the patient at the beginning.	4.9 (0.3)	3.9 (0.7)
Asked the patient one question at a time.	4.3 (0.8)	4.7 (0.9)
Listened to the patient without unnecessary interruption.	5.0 (0.0)	5.0 (0.0)
Asked questions to clarify his/her own understanding of the patient's answers.	4.7 (0.5)	4.0 (1.0)
Presented information at a pace that was easy to follow for both patient and interpreter; that is, information was given in "digestible chunks."	4.6 (0.7)	4.9 (0.3)
Maintained direct eye contact with the patient (instead of the interpreter).	4.9 (0.3)	4.5 (1.0)
Addressed the patient in the first person and not as "he/she."	4.9 (0.3)	4.8 (0.9)
Appropriately closed the encounter: At a minimum, the trainee asked the patient if he/she had any questions.	4.2 (0.6)	3.8 (0.4)
To what extent did the trainee keep the interpreter on track within his/her assigned role?	4.7 (0.5)	4.3 (0.5)
Global rating of trainee's effectiveness in using the interpreter for the patient encounter.	4.5 (0.5)	4.7 (0.5)

[a]Rated on a 5-point Likert scale (1 = *marginal/low,* 5 = *outstanding*).

Discussion

We designed and implemented two OSCE cases for residents utilizing SPs with LEP relying on an interpreter. The cases were well received by residents, certified medical interpreters, and SPs who reported that the cases accurately depicted real-life patient scenarios, including the personal and emotional contexts of illness. While these cases were designed as part of our yearlong, resident-led Spanish language curriculum, our findings suggest utility outside of the YRSI. Indeed, both YRSI

participants and nonparticipants found the OSCE realistic and useful. Additionally, residents from varied specialty training backgrounds successfully engaged with the OSCE.

As demonstrated on the assessments by interpreters, SPs, and the faculty observer, resident physicians lacked some basic communication skills for interacting with a non-English-speaking SP through an interpreter. These deficiencies highlight clear areas for learning that could be targeted in medical training. Emphasizing more effective body language and eye contact, educating residents on how to address patients' cultural beliefs, and explaining clearly the role of an interpreter could improve residents' readiness to work with interpreters and, by extension, their effectiveness in communicating with LEP patients during interpreter encounters.

These deficiencies have been previously documented in existing literature about physicians' effectiveness in medical encounters with LEP patients,[7,9,19] indicating the importance of improving medical training in this domain. There is reason for optimism as a recent study found that 76% of accredited medical schools in the US now report having some curricular material to teach medical students how to interact with LEP patients and/or use interpreters, though how this early exposure might translate to skills at the bedside later in training and practice is unclear.[20]

The checklists used for assessment were straightforward and required minimal advance training for interpreters, SPs, and faculty. While residents were pleased with the formative feedback they received, utility of the feedback to the residents might have been optimized had it been provided in real time. Since our evaluation was part of a study,[18] providing timely feedback was not possible for this project but could be easily worked into future iterations of the OSCE as a purely educational exercise. This is especially true of the case-specific checklists, since there was much room for improvement among residents. The case-specific checklists were lengthy and time intensive to complete and would likely be difficult to score in real time (as opposed to video review) simultaneously with the FORS. In future iterations, instead of deliberately scoring the case-specific checklists, educators could use them as feedback tools to highlight the psychosocial richness that may be uncovered through optimal patient-centered use of medical interpreters.

Finally, the significant difference in IIRS scores for resident participants in the abdominal pain versus back pain cases likely reflects individual differences in scoring style of specific SPs rather than a true difference in performance or difficulty between the cases. No differences between the cases were identified by the other checklists. Additionally, with participants scoring in the 4-5 range on the Likert scale for both cases, the significant difference found on the IIRS may not be educationally meaningful.

The measurement of effectiveness for this OSCE is limited by its size and scope. The findings were drawn from a small number of resident participants as this implementation served as a pilot project. Additionally, while we did ask residents to self-reflect on their growth in comfort interacting with non-English-speaking patients, it was beyond the scope of the project to assess whether having been exposed to these cases impacted the residents' practice behavior in real clinical settings. In addition, although the validated instruments we chose (IS, IIRS, FORS) were designed to be applicable to encounters with patients who speak any non-English language, we limited our SPs to native Spanish speakers, as part of our assessment for resident participants in our Spanish language learning course. Our findings may not be generalizable to the use of the case scenarios in other languages.

In the future, we hope to offer this OSCE to the entire YRSI group in tandem with small-group discussion sessions or self-directed learning on best practices in utilizing interpreters.[21] Given the straightforward nature of the medical topics contained in the OSCE, we plan to evaluate its appropriateness for use with medical students at our institution. We hope that these SP cases, if implemented as part of a broader curriculum, may allow for more effective education of graduate and undergraduate medical learners on interaction with LEP patients.

MedEdPORTAL®
The Journal of Teaching and Learning Resources

Emily Pinto Taylor, MD: Resident Physician, Department of Internal Medicine, Yale School of Medicine; Resident Physician, Department of Pediatrics, Yale School of Medicine

Arielle Mulenos: Postgraduate Research Associate, Department of Internal Medicine, Yale School of Medicine

Avik Chatterjee, MD, MPH: Physician, Boston Health Care for the Homeless Program; Instructor, Harvard Medical School; Associate Epidemiologist, Division of Global Health Equity, Department of Internal Medicine, Brigham and Women's Hospital

Jaideep S. Talwalkar, MD: Associate Professor, Department of Internal Medicine, Yale School of Medicine; Associate Professor, Department of Pediatrics, Yale School of Medicine

Acknowledgments
The authors are grateful for the assistance of the Yale School of Medicine's Standardized Patient Program, with the direct help of Barbara Hildebrand and Kali Cyrus, MD, in the recruiting of standardized patient actors and the creation of these cases.

Disclosures
None to report.

Funding/Support
None to report.

Prior Presentations
Mulenos A, Pinto Taylor E, Talwalkar JS. Partnering with interpreter services: a resident-driven approach to improve communication with LEP patients. Poster presented at: New England SGIM Regional Meeting; March 10, 2018; Boston, MA.

Ethical Approval
Reported as not applicable.

References

1. Di Blasi Z, Harkness E, Ernst E, Georgiou A, Kleijnen J. Influence of context effects on health outcomes: a systematic review. *Lancet.* 2001;357(9258):757-762. https://doi.org/10.1016/S0140-6736(00)04169-6
2. Stewart MA. Effective physician-patient communication and health outcomes: a review. *CMAJ.* 1995;152(9):1423-1433.
3. Timmins CL. The impact of language barriers on the health care of Latinos in the United States: a review of the literature and guidelines for practice. *J Midwifery Womens Health.* 2002;47(2):80-96. https://doi.org/10.1016/S1526-9523(02)00218-0
4. Flores G. The impact of medical interpreter services on the quality of health care: a systematic review.*Med Care Res Rev.* 2005;62(3):255-299. https://doi.org/10.1177/1077558705275416
5. Wilson E, Chen AH, Grumbach K, Wang F, Fernandez A. Effects of limited English proficiency and physician language on health care comprehension. *J Gen Intern Med.* 2005;20(9):800-806. https://doi.org/10.1111/j.1525-1497.2000174.x
6. Whatley M, Batalova J. Limited English proficient population of the United States. Migration Policy Institute website. https://www.migrationpolicy.org/article/limited-english-proficient-population-united-states-0#1. Published July 25, 2013. Accessed October 8, 2017.
7. Flores G, Laws MB, Mayo SJ, et al. Errors in medical interpretation and their potential clinical consequences in pediatric encounters. *Pediatrics.* 2003;111(1):6-14. https://doi.org/10.1542/peds.111.1.6
8. Adamo G. Simulated and standardized patients in OSCEs: achievements and challenges 1992-2003. *Med Teach.* 2003;25(3):262-270. https://doi.org/10.1080/0142159031000100300
9. Miller GE. The assessment of clinical skills/competence/performance. *Acad Med.* 1990:65(9)(suppl):S63-S67.
10. O'Rourke K, Gruener G, Quinones D, Stratta E, Howell J. Spanish bilingual medical student certification. *MedEdPORTAL.* 2013;9:9400. https://doi.org/15766/mep_2374-8265.9400
11. Callahan E, Garcia E, Rehm J. Talk louder? Communicating with your Spanish speaking patients. *MedEdPORTAL.* 2011;7:8427. https://doi.org/10.15766/mep_2374-8265.8427
12. Baker DW, Hayes R, Fortier JP. Interpreter use and satisfaction with interpersonal aspects of care for Spanish-speaking patients. *Med Care.* 1998;36(10):1461-1470. https://doi.org/10.1097/00005650-199810000-00004
13. Chatterjee A, Talwalkar JS. An innovative medical Spanish curriculum for resident doctors. *Med Educ.* 2012;46(5):521-522. https://doi.org/10.1111/j.1365-2923.2012.04261.x
14. Chatterjee A, Qin L, de la Paz García M, Talwalkar JS. Improving linguistic and cultural competence in the health sector: a medical Spanish curriculum for resident physicians. *J Span Lang Teach.* 2015;2(1):36-50. https://doi.org/10.1080/23247797.2015.1019288
15. Gottfried S, Talwalkar JS. Optimizing the involvement of language interpreters during the clinical encounter. *J Gen Intern Med.* 2014;29(6):835. https://doi.org/10.1007/s11606-014-2858-1

16. Lie D, Bereknyei S, Braddock CH III, Encinas J, Ahearn S, Boker JR. Assessing medical students' skills in working with interpreters during patient encounters: a validation study of the Interpreter Scale. *Acad Med.* 2009;84(5):643-650. https://doi.org/10.1097/ACM.0b013e31819faec8
17. Lie D, Boker J, Bereknyei S, Ahearn S, Fesko C, Lenahan P. Validating measures of third year medical students' use of interpreters by standardized patients and faculty observers. *J Gen Intern Med.* 2007;22(suppl 2):336-340. https://doi.org/10.1007/s11606-007-0349-3
18. Mulenos A, Pinto Taylor E, Talwalkar JS. Partnering with interpreter services: a resident-driven approach to improve communication with LEP patients. Poster presented at: New England SGIM Regional Meeting; March 10, 2018; Boston, MA.
19. Brooks K, Stifani B, Batlle HR, Nunez MA, Erlich M, Diaz J. Patient perspectives on the need for and barriers to professional medical interpretation. *R I Med J.* 2016;99(1):30-33.
20. Himmelstein J, Wright WS, Wiederman MW. U.S. medical school curricula on working with medical interpreters and/or patients with limited English proficiency. *Adv Med Educ Pract.* 2018;9:729-733. https://doi.org/10.2147/AMEP.S176028
21. Gottfried S. Language support services. In: Talwalkar JS, Fenick AM, eds. *Yale Primary Care Pediatrics Curriculum: Version 12.* New Haven, CT: Yale Printing and Publishing Services; 2018.

Received: November 13, 2018 | **Accepted:** April 8, 2019 | **Published:** May 20, 2019
