# Supplementary material for: Partnering With Interpreter Services: Standardized Patient Cases to Improve Communication With Limited English Proficiency Patients
Source: MedEdPORTAL. 2019 May 20;15:10826. doi: 10.15766/mep_2374-8265.10826 (PMC6543860; doi:10.15766/mep_2374-8265.10826)
Supplement: Supplementary file 1 — A. Case 1 SP Information.docx B. Case 2 SP Information.docx C. Case 1 Resident Participant Information.docx D. Case 2 Resident Participant Information.docx E. Case 1 Physical Exam Sheet.docx F. Case 2 Physical Exam Sheet.docx G. UCI Interpreter Scale.docx H. UCI Interpreter Impact Rating Scale.docx I. Resident Session Evaluation Form.docx J. OSCE Workshop Schedule.docx K. UCI FORS Scale.docx L. Case 1 Observer Checklist.xlsx M. Case 2 Observer Checklist.xlsx [file mep-15-10826-s001.zip › A. Case 1 SP Information.docx]

Appendix A: MedEdPORTAL Standardized Patient Case Development Tool

Date: March 2, 2019

Primary Case Author: Dr. Emily Pinto Taylor

Secondary Case Author: Arielle Mulenos, Dr. Jaideep Talwalkar

Standardized Patient Educator: Dr. Kali Cyrus

Name of Case: Abdominal Pain

Name of educational and or assessment activity: Using an Interpreter

Patient Name: Eliana/Elian Rodriguez

Chief Complaint: Abdominal Pain

Most likely Diagnosis and Differential with rationale from history and/or physical exam:

DDX: gastritis, peptic ulcer disease, cholecystitis, biliary colic, appendicitis, and ectopic pregnancy/ovarian torsion (if female). Most likely diagnosis appendicitis, with sudden onset abdominal pain, nausea, anorexia, epigastric tenderness that migrated to RLQ with voluntary guarding.

Challenge question: N/A

Domains: Check all that apply

- Professionalism

X Communication and Interpersonal skills

X Medical History

- Physical exam
- Shared Decision Making
- Patient Education
- Clinical Reasoning
- Documentation
- Handoff
- Presentation
- Other:

Type and level of learner: Resident Physician

Case Objectives: please list specific objectives for each of the domains you have checked above:

1. Communication and interpersonal skills:
   1. By the end of this activity, learners will be able to:
      1. identify the role of the interpreter in a clinical encounter.
      2. demonstrate best practices in working with an interpreter, such as speaking in short phrases, using second-person, and minimizing medical jargon.
2. Medical History:
   1. By the end of this activity, learners will be able to utilize an interpreter to obtain a history from a Spanish-speaking standardized patient.

| SETTING: outpatient, in patient, ED, home, nursing home, rehab, group etc. | Outpatient urgent care clinic |
| --- | --- |
| PATIENT PROFILE: Information about the “patient” that helps select an SP and helps the learner get an understanding of them as a person. SP will know more information about the patient than learner will ever ask but allows SP to portray a fully developed patient personality. If none of the items below are particulars for the case please write “all may be used.” | |
| Age range | 25-35 years old |
| Religious/spiritual background | All may be used |
| Sex (e.g., male, female, intersex, transwoman, transman) | Female/Male |
| Sexual Orientation (e.g., heterosexual, lesbian, gay, bisexual, pansexual, queer, asexual) | All may be used |
| Gender expression (e.g., man, woman, gender queer) | All may be used |
| Race/ethnicity: | All may be used |
| Physical description (e.g., BMI, height range) | All may be used |
| Physical limitations | All may be used |
| Patient appearance (e.g., disheveled, hospital gown, business casual, casual) | All may be used |
| Moulage + location (e.g., none, bruises, scars, body piercing, tattoos) | All may be used |
| Affect (e.g., pleasant, cooperative) | Pleasant, uncomfortable |
| Family group (e.g., who is family, who they live with) | You live with a roommate in a small city.  The remainder of your family lives in your home country. This includes three older sisters and a mother, who is in her 60s. Your father passed away two years ago when he was 66. |
| Education | All may be used |
| Level of health literacy | Average |
| Employment, if any - present and past, noting any current stresses | You recently obtained a job as assistant chef in a local restaurant. You had been working odd jobs in that kitchen up until now. |
| Home/homeless - type of dwelling, number of stories, owned or rented | You live with a roommate in a small city. |
| Financial situation- any current stresses | You recently obtained a job which allows you to comfortably pay rent and stay in the country. Your mother would like you to return to your country of origin, and you are upset about the strain on your relationship. You are upset about the abdominal pain because you can’t afford to miss work. |
| Insurance Status (e.g., un/under/insured, public/private, HMO/PPO) | All may be used |
| Habits (i.e., diet, exercise, caffeine, smoking, alcohol, drugs) | You drink alcohol once or twice a week, one-two drinks, but have not had a drink in the past week.  You smoked cigarettes for a brief stretch (few weeks) while in school back home, but you haven’t smoked since.  You do not use any drugs. |
| Activities (i.e., hobbies, sports, clubs, friends) | All may be used |
| Typical day - what is the usual daily routine | All may be used |

| CASE INFORMATION | |
| --- | --- |
| Chief Concern: What the patient will say when greeted by the student. The patient’s primary reason for seeking medical care often stated in his/own words. | “My stomach hurts” |
| Additional Concerns: Other, if any, concerns the patient has today (i.e., symptoms, requests, expectations, etc.) that will become part of set agenda. | N/A |
|  | |
| THE PATIENT STORY: The SP will be asked to tell their symptom story and the personal and emotion impact for each of their concerns. You will want to write this is the patient voice. The symptom story should be able to answer this question: “Tell me more about [chief concern/additional concern], starting at the beginning and bringing me up to now.”  The personal context should be able to answer questions concerning the broader personal/psychosocial context of symptoms, especially the patient beliefs/attributions.  The emotional context should be able to ask how are you doing with this, how does this make you feel, how has this affected you emotionally? IMPACT: How has this affected your life? How has this been for your family? | You are a 25-year-old with abdominal pain since last night. The pain started last night and was 5/10 in severity. It started in the upper, central part of the stomach and was very severe, sharp and throbbing in quality. You tried some over-the-counter antacids (TUMS) without relief. You couldn’t sleep because of the pain, and started developing nausea. You started vomiting around 4 am, and have vomited twice without relief of the abdominal pain. No blood in the vomit—it just looks like food and clear liquid. A low-grade fever started this morning bit you haven’t checked your temperature with a thermometer. A few hours ago, the pain moved into the right lower part of your abdomen. The pain is now 6/10. No diarrhea, no constipation, no blood in your stool. You have lost your appetite.  No pain with urination or other urinary symptoms. No headache, no sore throat, no cough, no chest pain or shortness of breath, no rashes, no other symptoms. No recent trauma, unusual meals, or travel outside the local area. You are not aware of any friends, family members, or coworkers with similar symptoms.  You are scared and worried. You feel these emotions because you worry that this abdominal pain might be something serious and you can't afford to miss work. You recently got this job, which is allowing you to comfortably pay rent and stay in the USA. Your mother has been insisting that you return to your country of origin. Things are going really well with your significant other and you don't want to leave. |
| HISTORY OF PRESENT ILLNESS: Although some of the HPI will be given in the patient’s symptom story, the learners will expand the story during the direct question section. Below describe the detailed history, usually about the chief concern, which the student must develop in order to make a useful assessment of the problem: | |
|  | |
| Onset (when; gradual or sudden) | The pain started last night and was 5/10 in severity. The pain is now 6/10 in severity. |
| Setting (what was going on or where was patient when symptoms first noticed?) | N/A |
| Duration (how long) | The pain began last night with 5/10 severity.  Vomiting occurred twice beginning at 4 am.  Low-grade fever started this morning. The pain is now at 6/10. |
| Time relationships (frequency, constant or intermittent) | Pain is constant and increasing in severity. |
| Location | Pain started in the upper, central part of the stomach and was very severe, sharp and throbbing in quality.   A few hours ago, the pain moved into the right lower part of your abdomen. |
| Radiation | Absent |
| Quality | The pain is very severe, sharp and throbbing in quality |
| Amount | The 5/10 pain severity began last night, but has increased to 6/10 as of this morning. |
| Aggravated by what | N/A |
| Relieved by what | N/A |
| Associated with what | Unsure |
| Attitude (what does the patient think is the problem, and how does he/she feel about it) | You are scared and worried. You worry that this abdominal pain might be something serious and you can't afford to miss work. |
| Overall course | Pain is worsening overall, and nausea has developed. |
| REVIEW OF SYSTEMS: Significant positives and negatives | |
|  | Review of systems is negative. |
|  |  |
|  |  |
|  |  |
|  | |
| Past medical history |  |
| Medication allergies (Name and reaction) | Penicillin (got a rash as a child when taken for a sore throat) |
| Environmental allergies (Name and reaction) | N/A |
| Illnesses | History of asthma as a child, but you have not needed to use your albuterol inhaler for three years  Mild skin infection two months ago. An antibiotic was prescribed, but you can’t recall which one. |
| Vaccinations | Up to date with routine vaccinations |
| Surgeries | No abdominal surgeries |
| Accidents/ injuries/ trauma | None |
| Hospitalization | None |
|  | |
| Inclusive sexual and reproductive history | |
| Sexual practices  Sexual partners  Protection: Use of safer sex practices  Use of birth control if appropriate  Risk of intimate partner violence | You are sexually active with your girl/boyfriend, monogamous, of the past nine months.  You use condoms most of the time. You are on daily oral contraceptives (name brand Ortho Tri-Cyclen or similar) if female actor. |
| Ob/GYN HISTORY | Age of onset of menses: 14-years-old  Age of menopause: N/A  Number of pregnancies: 0  Number of live births: 0  Number of miscarriages: 0  Number of abortions: 0 |
| Medications | Prescription/dose/reason: Ortho-Tri-Cyclen for pregnancy prevention (if female)  Over the counter/dose/reason: N/A  Herbs/supplements/dose/reason: N/A  Other: N/A |
| Immunizations | X Tetanus   - Flu - Hepatitis - Pneumovax - HPV - Other |
| Tobacco products:   - Cigarettes - Cigar - Pipe - Chew - E-cigarettes | X Never   - Past- year started/year quit - Current   - Quantity   - # of years |
| Alcohol   - Beer - Wine - Liquor - Other | - Never - Past- year started/year quit   X Current   - - Quantity: 1-2 drinks per week   - # of years: N/A |
| Drugs   - Weed - Cocaine - Heroin - Meth - Other - IV - Inhalants - Other | X Never   - Past- year started/year quit - Current   - Quantity - # of years |
| Diet (describe) | N/A |
| Exercise (describe) | N/A |
| List any other important social history or information important to this case | N/A |
| Family history |  |
| Mother, Father, Siblings, Grandparents, and other significant findings. | Your mother is in her 60s with high blood pressure and diabetes.  Your father passed away of a heart attack when he was 66 (two years ago).  Three older sisters are all healthy and live in your country of origin. |
|  |  |
| Physical Exam- List exam maneuvers expected for this case and any abnormal findings that SP will simulate. (tenderness, hyper-hypo reflex, rebound, weakness etc. )  No physical exam performed, participant will receive a slip of paper with documented exam findings (Appendix E). | |
| PHYSICAL EXAM FINDINGS |  |
| 1. Written in layman’s terms | **General:** Mild distress lying on the exam table  **CV:** Regular rhythm, fast heart rate, no murmur  **Pulm:** Clear on both sides  **Abd:** Bowel sounds present, pain present all over with palpation, most tender in right lower quadrant  **Extr:** warm, good blood flow  **Urine pregnancy test is negative (if this is a female patient)** |
| 1. General appearance- affect, appearance, position of patient at opening (i.e. sitting, laying down, holding abdomen etc.) | Sitting on exam table, conversational, mildly uncomfortable with changing position on table. Should be sitting on the exam table, seated and looking at trainee. |
| 1. Vital signs | (Given prior to entering the patient room)  Temperature: 100° F  Heart Rate: 105 beats per minute  Respiration Rate: 18 breaths per minute  Oxygen Saturation: 99% on room air |
| 1. Specific findings and affect | N/A |
| 1. Response to certain physical movements | N/A |
|  |  |
| DIAGNOSIS AND DIFFERENTIAL |  |
| Diagnosis with support from positive and negative history and PE findings | Appendicitis, given sudden onset abdominal pain, nausea, anorexia, epigastric tenderness that migrated to RLQ with voluntary guarding. Importantly, no pertinent sexual history, menstrual history, or recurrence of prior pain. |
| Differential with support from positive and negative history and PE findings | Gastritis, peptic ulcer disease, cholecystitis, biliary colic, appendicitis, and ectopic pregnancy/ovarian torsion (if female). |
|  |  |
| MANAGEMENT OR DIAGNOSTIC PLAN | Abdominal imaging (ultrasound or CT scan)  Nothing to be consumed by mouth until results of scan Pain control (NSAIDs, prefer to avoid opiates)  Antiemetic |
|  |  |
| PROFESSIONALISM ISSUES OR CHALLENGES: | Use of interpreter throughout the encounter, with special attention played to position of chairs, location of patient, interpreter, and resident interviewee. Will also be important to observe use of second-person to address patient, length of phrases prior to allowing interpreter to speak, and cultural sensitivity of resident physician participant. |
