# Supplementary material for: Partnering With Interpreter Services: Standardized Patient Cases to Improve Communication With Limited English Proficiency Patients
Source: MedEdPORTAL. 2019 May 20;15:10826. doi: 10.15766/mep_2374-8265.10826 (PMC6543860; doi:10.15766/mep_2374-8265.10826)
Supplement: Supplementary file 1 — A. Case 1 SP Information.docx B. Case 2 SP Information.docx C. Case 1 Resident Participant Information.docx D. Case 2 Resident Participant Information.docx E. Case 1 Physical Exam Sheet.docx F. Case 2 Physical Exam Sheet.docx G. UCI Interpreter Scale.docx H. UCI Interpreter Impact Rating Scale.docx I. Resident Session Evaluation Form.docx J. OSCE Workshop Schedule.docx K. UCI FORS Scale.docx L. Case 1 Observer Checklist.xlsx M. Case 2 Observer Checklist.xlsx [file mep-15-10826-s001.zip › B. Case 2 SP Information.docx]

Appendix A: MedEdPORTAL Standardized Patient Case Development Tool

Date: March 2, 2019

Primary Case Author: Emily Pinto Taylor

Secondary Case Author: Arielle Mulenos, Dr. Jaideep Talwalkar

Standardized Patient Educator: Dr. Kali Cyrus

Name of Case: Low Back Pain

Name of educational and or assessment activity: Using an Interpreter

Patient Name: Pedro/Ana Gonzales

Chief Complaint: “My back hurts”

Most likely Diagnosis and Differential with rationale from history and/or physical exam:

DDX: Sciatica, vertebral fracture, lumbar disc herniation with radiculopathy, lumbar paraspinal muscle strain.

Most likely diagnosis: Lumbar paraspinal muscle strain, with trauma causing “wrenching” sensation, relief with heat/NSAIDs, but minimal improvement with rest, worse with exertion, and no neurological symptoms. Exam with negative straight-leg raise but paraspinal muscle tenderness to palpation. No bony tenderness noted.

Challenge question:

Domains: Check all that apply

- Professionalism

X Communication and Interpersonal skills

X Medical History

- Physical exam
- Shared Decision Making
- Patient Education
- Clinical Reasoning
- Documentation
- Handoff
- Presentation
- Other:

Type and level of learner: Resident Physician

Case Objectives: please list specific objectives for each of the domains you have checked above:

1. Communication and interpersonal skills:
   1. By the end of this activity, learners will be able to:
      1. identify the role of the interpreter in a clinical encounter.
      2. consider best practices in working with an interpreter, such as speaking in short phrases, using second-person, and minimizing medical jargon.
2. Medical History:
   1. By the end of this activity, learners will be able to utilize an interpreter to obtain a history from a Spanish-speaking standardized patient.

| SETTING: outpatient, in patient, ED, home, nursing home, rehab, group etc. | Outpatient Urgent Care Clinic |
| --- | --- |
| PATIENT PROFILE: Information about the “patient” that helps select an SP and helps the learner get an understanding of them as a person. SP will know more information about the patient than learner will ever ask but allows SP to portray a fully developed patient personality. If none of the items below are particulars for the case please write “all may be used.” | |
| Age range | 30-years-old |
| Religious/spiritual background | All may be used |
| Sex (e.g., male, female, intersex, transwoman, transman) | Male or Female |
| Sexual Orientation (e.g., heterosexual, lesbian, gay, bisexual, pansexual, queer, asexual) | All may be used |
| Gender expression (e.g., man, woman, gender queer) | All may be used |
| Race/ethnicity: | All may be used |
| Physical description (e.g., BMI, height range) | All may be used |
| Physical limitations | Low back pain worsened with physical activity (riding a bike). |
| Patient appearance (e.g., disheveled, hospital gown, business casual, casual) | All may be used |
| Moulage + location (e.g., none, bruises, scars, body piercing, tattoos) | All may be used |
| Affect (e.g., pleasant, cooperative) | Pleasant, uncomfortable |
| Family group (e.g., who is family, who they live with) | You live with your significant other and two kids, ages seven and three. |
| Education | All may be used |
| Level of health literacy | Average |
| Employment, if any - present and past, noting any current stresses | You work in the warehouse of a big-box store. |
| Home/homeless - type of dwelling, number of stories, owned or rented | You live with your significant other and two kids, ages seven and three. |
| Financial situation- any current stresses | You are the sole income-earner in the household. Losing your job would mean losing your medical insurance and would make it hard to support your family. |
| Insurance Status (e.g., un/under/insured, public/private, HMO/PPO) | Recently insured through work. Was previously uninsured for years. |
| Habits (i.e., diet, exercise, caffeine, smoking, alcohol, drugs) | Alcohol: 1-2 beers, 3-4 times per week Tobacco: a few cigarettes daily, more with alcohol  Marijuana: once per month or so  Cocaine: tried once, 10 years ago |
| Activities (i.e., hobbies, sports, clubs, friends) | All may be used |
| Typical day - what is the usual daily routine | All may be used |

| CASE INFORMATION | |
| --- | --- |
| Chief Concern: What the patient will say when greeted by the student. The patient’s primary reason for seeking medical care often stated in his/own words. | “My back hurts” |
| Additional Concerns: Other, if any, concerns the patient has today (i.e., symptoms, requests, expectations, etc.) that will become part of set agenda. | N/A |
|  | |
| THE PATIENT STORY: The SP will be asked to tell their symptom story and the personal and emotion impact for each of their concerns. You will want to write this is the patient voice. The symptom story should be able to answer this question: “Tell me more about [chief concern/additional concern], starting at the beginning and bringing me up to now.”  The personal context should be able to answer questions concerning the broader personal/psychosocial context of symptoms, especially the patient beliefs/attributions.  The emotional context should be able to ask how are you doing with this, how does this make you feel, how has this affected you emotionally? IMPACT: How has this affected your life? How has this been for your family? | You are a 30-year-old with 2 days of new low back pain, much worse than anything you’ve had before. You were helping a friend move out of his apartment two days ago and felt a wrenching pain in your back. The pain was 6/10 and you tried some ibuprofen for the pain with minimal relief. A heating pad helped slightly while it was on. You also spent all day yesterday in bed without relief of the pain.  The pain comes in spasms. The pain is in your lower back, worse on the right side, and does not spread anywhere else.  You were able to sleep at night but came in because the pain has not gotten better.  No trouble walking, though you tried to ride your bike into work this morning and the pain became very severe. You have no symptoms in your legs like numbness, tingling, or weakness.  No problems with urination or defecation, no numbness in the groin area.  In the past, you have had episodes of mild back pain that have gone away quickly without any specific treatment, but none quite this bad.  No fever, no headache, no abdominal pain, vomiting, diarrhea, or constipation. No other symptoms.  You are scared and worried. You are worried about losing your job, which requires heavy lifting. You just managed to get health insurance through your work, after having been uninsured for years. Your 3-year-old son has severe behavioral problems and you were finally able to get the medical appointments he needed. Your significant other has been unable to work because you couldn't find a childcare that would take your 3-year-old son since he is so hard to manage. You are the sole income-earner in the household. Losing your job would mean losing your insurance and would make it hard to support your family. |
| HISTORY OF PRESENT ILLNESS: Although some of the HPI will be given in the patient’s symptom story, the learners will expand the story during the direct question section. Below describe the detailed history, usually about the chief concern, which the student must develop in order to make a useful assessment of the problem: | |
|  | |
| Onset (when; gradual or sudden) | Pain started two days ago suddenly when you were helping a friend move out of his apartment. You felt a wrenching pain in your back at a pain level of 6/10. |
| Setting (what was going on or where was patient when symptoms first noticed?) | You were helping a friend move out of his apartment when you felt a wrenching pain in your back. |
| Duration (how long) | Two days |
| Time relationships (frequency, constant or intermittent) | Pain comes in spasms |
| Location | The pain is in your lower back, worse on the right side, and does not spread anywhere else. |
| Radiation | Absent |
| Quality | Twisting, spasming |
| Amount | 6/10 in severity |
| Aggravated by what | Riding your bike made the pain very severe. |
| Relieved by what | Ibuprofen provided minimal relief. A heating pad helped slightly while it was on. |
| Associated with what | Pain worsens with physical activity such as riding a bike. |
| Attitude (what does the patient think is the problem, and how does he/she feel about it) | You are scared and worried. You are worried about losing your job, which requires heavy lifting. |
| Overall course | Pain continues in spasms without much relief. |
| REVIEW OF SYSTEMS: Significant positives and negatives | |
|  | Review of systems is negative |
|  | |
| Past medical history |  |
| Medication allergies (Name and reaction) | Penicillin (got a rash once as a child) |
| Environmental allergies (Name and reaction) | N/A |
| Illnesses | History of eczema as a child, but that resolved.  Several episodes of ear infections as a child |
| Vaccinations | Up to date |
| Surgeries | None |
| Accidents/ injuries/ trauma | You broke your left arm as a child in a fall |
| Hospitalization | None |
|  | |
| Inclusive sexual and reproductive history | |
| Sexual practices  Sexual partners  Protection: Use of safer sex practices  Use of birth control if appropriate  Risk of intimate partner violence | You are monogamous with your significant other. (If female): You have a Mirena IUD, which you use for contraception. You don’t get periods any longer since the Mirena was placed. |
| OB/GYN History (if female) | Age of onset of menses: 14, as above, no longer getting periods with IUD  Age of menopause: N/A  Number of pregnancies: 2  Number of live births: 2  Number of miscarriages: 0  Number of abortions: 0 |
| Medications | Prescription/dose/reason: None  Over the counter/dose/reason: Ibuprofen (two pills) as needed for pain. You do not know the milligram strength.  Herbs/supplements/dose/reason: None |
| Immunizations | X Tetanus   - Flu - Hepatitis - Pneumovax - HPV - Other |
| Tobacco products:  X Cigarettes   - Cigar - Pipe - Chew - E-cigarettes | - Never - Past- year started/year quit   X Current   - - Quantity: A few cigarettes a day; more when you drink   - # of years: “a long time” |
| Alcohol  X Beer   - Wine - Liquor - Other | - Never - Past- year started/year quit   X Current   - - Quantity: 1-2 beers, three or four times a week |
| Drugs  X Marijuana  X Cocaine   - Heroin - Meth - Other - IV - Inhalants - Other | - Never   X Past- year started/year quit: Cocaine - tried once 10 years ago  X Current: Marijuana   - - Marijuana Quantity: Once a month |
| Diet (describe) | N/A |
| Exercise (describe) | N/A |
| List any other important social history or information important to this case | N/A |
| Family history |  |
| Mother, Father, Siblings, Grandparents, and other significant findings. | Mother has high blood pressure and arthritis.  Father has diabetes and kidney problems (you do not know details).  Your parents still live in your country of origin. |
|  |  |
| Physical Exam- List exam maneuvers expected for this case and any abnormal findings that SP will simulate. (tenderness, hyper-hypo reflex, rebound, weakness etc. )  No physical exam will be performed, SP will provide exam findings on a piece of paper to resident physician when prompted (Appendix F). | |
| PHYSICAL EXAM FINDINGS |  |
| 1. Written in layman’s terms | General: No acute distress, mild discomfort when changing position.  Abdominal: Bowel sounds present, not tender.  Back: Normal to inspection, full range of motion. No tenderness over spine and most muscles, except muscles on low back, right side. No pain when legs are raised on either side.  Neurological: Normal reflexes, strength and sensation in lower extremities on both sides.  Extremities: Warm with good blood flow |
| 1. General appearance- affect, appearance, position of patient at opening (i.e. sitting, laying down, holding abdomen etc.) | Sitting on exam table, conversational, mildly uncomfortable with changing position on table. Should be seated on exam table and looking at trainee. |
| 1. Vital signs | (Provided to resident physician prior to entering the patient room)  Temperature: 98.5° F  Heart Rate: 80 beats per minute  Respiration Rate: 18 breaths per minute  Oxygen Saturation: 99% on room air |
| 1. Specific findings and affect | N/A |
| 1. Response to certain physical movements | N/A |
|  |  |
| DIAGNOSIS AND DIFFERENTIAL |  |
| Diagnosis with support from positive and negative history and PE findings | Most likely diagnosis: Lumbar paraspinal muscle strain, with trauma causing “wrenching” sensation, relief with heat/NSAIDs, but minimal improvement with rest, worse with exertion, and no neurological symptoms. Exam with negative straight-leg raise but paraspinal muscle tenderness to palpation. No bony tenderness noted. |
| Differential with support from positive and negative history and PE findings | DDX: Sciatica, vertebral fracture, lumbar disc herniation with radiculopathy, lumbar paraspinal muscle strain. |
|  |  |
| MANAGEMENT OR DIAGNOSITIC PLAN | Rest, ice/heat, stretching NSAIDs for pain relief, could consider muscle relaxant for a few days if unable to stretch due to pain  Physical therapy if no improvement with conservative treatment  Return for follow up |
|  |  |
| PROFESSIONALISM ISSUES OR CHALLENGES: | Use of interpreter throughout the encounter, with special attention played to position of chairs, location of patient, interpreter, and resident interviewee. Will also be important to observe use of second-person to address patient, length of phrases prior to allowing interpreter to speak, and cultural sensitivity of resident physician participant. |
