# Supplementary material for: Partnering With Interpreter Services: Standardized Patient Cases to Improve Communication With Limited English Proficiency Patients
Source: MedEdPORTAL. 2019 May 20;15:10826. doi: 10.15766/mep_2374-8265.10826 (PMC6543860; doi:10.15766/mep_2374-8265.10826)
Supplement: Supplementary file 1 — A. Case 1 SP Information.docx B. Case 2 SP Information.docx C. Case 1 Resident Participant Information.docx D. Case 2 Resident Participant Information.docx E. Case 1 Physical Exam Sheet.docx F. Case 2 Physical Exam Sheet.docx G. UCI Interpreter Scale.docx H. UCI Interpreter Impact Rating Scale.docx I. Resident Session Evaluation Form.docx J. OSCE Workshop Schedule.docx K. UCI FORS Scale.docx L. Case 1 Observer Checklist.xlsx M. Case 2 Observer Checklist.xlsx [file mep-15-10826-s001.zip › C. Case 1 Resident Participant Information.docx]

Appendix C – Case 1, Resident Instruction Sheet

**Information for Physician**

**Abdominal Pain**

You are moonlighting in an urgent care clinic. (Insert Name) is your first patient. The triage sheet indicates a 25-year-old with a chief concern of abdominal pain. The patient is exclusively Spanish-speaking. The medical assistant has already called an interpreter. Vital signs obtained by the nurse are:

Temp 100.0, HR 105, RR 18, O2 sat 99% on RA

You will have 15 minutes for this visit. Obtain a history. After you have completed your history, review the results of the physical exam which will be in a folder in the room - you will not be performing a physical exam. Only review the physical exam folder AFTER you are done with the history. Counsel the patient about your care plan before completing the visit.
