# Supplementary material for: Partnering With Interpreter Services: Standardized Patient Cases to Improve Communication With Limited English Proficiency Patients
Source: MedEdPORTAL. 2019 May 20;15:10826. doi: 10.15766/mep_2374-8265.10826 (PMC6543860; doi:10.15766/mep_2374-8265.10826)
Supplement: Supplementary file 1 — A. Case 1 SP Information.docx B. Case 2 SP Information.docx C. Case 1 Resident Participant Information.docx D. Case 2 Resident Participant Information.docx E. Case 1 Physical Exam Sheet.docx F. Case 2 Physical Exam Sheet.docx G. UCI Interpreter Scale.docx H. UCI Interpreter Impact Rating Scale.docx I. Resident Session Evaluation Form.docx J. OSCE Workshop Schedule.docx K. UCI FORS Scale.docx L. Case 1 Observer Checklist.xlsx M. Case 2 Observer Checklist.xlsx [file mep-15-10826-s001.zip › E. Case 1 Physical Exam Sheet.docx]

Appendix E – Case 1, Physical Exam Information Sheet

**Abdominal Pain: Physical Exam Information**

**General:** Mild distress lying on the exam table

**CV:** Regular, tachycardic, no murmurs

**Pulm:** Clear to auscultation bilaterally

**Abd:** Bowel sounds present, voluntary guarding present, tender to palpation in right lower quadrant, no rebound present, equivocal Rovsing's sign, negative psoas and obturator signs

**Extr:** warm and well-perfused

**Urine pregnancy test is negative (if this is a female patient)**
