# Supplementary material for: Partnering With Interpreter Services: Standardized Patient Cases to Improve Communication With Limited English Proficiency Patients
Source: MedEdPORTAL. 2019 May 20;15:10826. doi: 10.15766/mep_2374-8265.10826 (PMC6543860; doi:10.15766/mep_2374-8265.10826)
Supplement: Supplementary file 1 — A. Case 1 SP Information.docx B. Case 2 SP Information.docx C. Case 1 Resident Participant Information.docx D. Case 2 Resident Participant Information.docx E. Case 1 Physical Exam Sheet.docx F. Case 2 Physical Exam Sheet.docx G. UCI Interpreter Scale.docx H. UCI Interpreter Impact Rating Scale.docx I. Resident Session Evaluation Form.docx J. OSCE Workshop Schedule.docx K. UCI FORS Scale.docx L. Case 1 Observer Checklist.xlsx M. Case 2 Observer Checklist.xlsx [file mep-15-10826-s001.zip › F. Case 2 Physical Exam Sheet.docx]

Appendix F – Case 2, Physical Exam Information Sheet

**Back Pain: Physical Exam Information**

**General:** No acute distress, mild discomfort when changing position

**Abd:** Bowel sounds present, non-tender

**Back:** Normal to inspection, full range of motion, no tenderness over spinous processes, tenderness over lumbar paraspinal muscles, right more than left, negative straight leg raises bilaterally

**Neuro:** Normal reflexes, strength and sensation in lower extremities bilaterally

**Extr:** warm and well-perfused
