# Supplementary material for: Partnering With Interpreter Services: Standardized Patient Cases to Improve Communication With Limited English Proficiency Patients
Source: MedEdPORTAL. 2019 May 20;15:10826. doi: 10.15766/mep_2374-8265.10826 (PMC6543860; doi:10.15766/mep_2374-8265.10826)
Supplement: Supplementary file 1 — A. Case 1 SP Information.docx B. Case 2 SP Information.docx C. Case 1 Resident Participant Information.docx D. Case 2 Resident Participant Information.docx E. Case 1 Physical Exam Sheet.docx F. Case 2 Physical Exam Sheet.docx G. UCI Interpreter Scale.docx H. UCI Interpreter Impact Rating Scale.docx I. Resident Session Evaluation Form.docx J. OSCE Workshop Schedule.docx K. UCI FORS Scale.docx L. Case 1 Observer Checklist.xlsx M. Case 2 Observer Checklist.xlsx [file mep-15-10826-s001.zip › G. UCI Interpreter Scale.docx]

UCI School of Medicine
Interpreter Checklist (IS)

**Checklist for Interpreter to complete after each encounter**

Trainee’s Name: __________________ Date: ________________________

Language of Encounter: ____________

Interpreter’s Name: _______________ Interpreter (circle one): Professional / Standardized

Interpreter to score trainee as **Done** or **Not Done** or **Circle** appropriate number.

|  |  | **Done** | **Not Done** |
| --- | --- | --- | --- |
| 1 | The trainee introduced himself or herself to me. |  |  |
| 2 | The trainee introduced me to the patient. |  |  |
| 3 | The trainee adequately explained the purpose of the interview. | 1 2 3 4 5 Poor Outstanding | |
| 4 | The trainee explained my role to the patient at the beginning. | 1 2 3 4 5 Poor Outstanding | |
| 5 | The trainee arranged the seating in a manner conductive to effective interpretation. | 1 2 3 4 5 Poor Outstanding | |
| 6 | The trainee asked me one question at a time. | 1 2 3 4 5 Poor Outstanding | |
| 7 | The trainee listened to me as I interpreted the patient’s answers, without unnecessary interruption. | 1 2 3 4 5 Poor Outstanding | |
| 8 | The trainee asked questions to clarify his/her own understanding of the patient’s answers. | 1 2 3 4 5 Poor Outstanding | |
| 9 | The trainee asked the patient if he or she had any questions. | 1 2 3 4 5 Poor Outstanding | |
| 10 | The trainee maintained direct eye contact with the patient instead of me most of the time. | 1 2 3 4 5 Poor Outstanding | |
| 11 | The trainee addressed the patient in first person, and not as “he/she.” | 1 2 3 4 5 Poor Outstanding | |
| 12 | The trainee kept me “on track.” (eg: questioned me when lapses led to incomplete translations) | 1 2 3 4 5 Poor Outstanding | |
| 13 | Rate your overall satisfaction with the encounter. | 1 2 3 4 5 Poor Outstanding | |

**Interpreter Comments:**

Please describe specific trainee behaviors that either enhanced or detracted from the effectiveness of your interpretation and connecting with the patient during the encounter (EXPLANATORY NOTE: Please describe how the trainee’s behavior made you feel (either positively or negatively). Balance all constructive criticism with praise for the things the trainee did well).
