# Supplementary material for: Partnering With Interpreter Services: Standardized Patient Cases to Improve Communication With Limited English Proficiency Patients
Source: MedEdPORTAL. 2019 May 20;15:10826. doi: 10.15766/mep_2374-8265.10826 (PMC6543860; doi:10.15766/mep_2374-8265.10826)
Supplement: Supplementary file 1 — A. Case 1 SP Information.docx B. Case 2 SP Information.docx C. Case 1 Resident Participant Information.docx D. Case 2 Resident Participant Information.docx E. Case 1 Physical Exam Sheet.docx F. Case 2 Physical Exam Sheet.docx G. UCI Interpreter Scale.docx H. UCI Interpreter Impact Rating Scale.docx I. Resident Session Evaluation Form.docx J. OSCE Workshop Schedule.docx K. UCI FORS Scale.docx L. Case 1 Observer Checklist.xlsx M. Case 2 Observer Checklist.xlsx [file mep-15-10826-s001.zip › H. UCI Interpreter Impact Rating Scale.docx]

UCI School of Medicine
Interpreter Impact Rating Scale (IIRS)

Checklist for **Standardized Patient** to complete on trainee after each encounter

Trainee’s Name: __________________ SP’s Name: ________________________

How did the presence and involvement of the interpreter affect the interaction between the patient and the trainee? Please rate the following items.

|  |  | **Outstanding** | **Very good** | **Good** | **Needs Improvement** | **Marginal** |
| --- | --- | --- | --- | --- | --- | --- |
| 1 | The trainee showed direct eye contact with me during the encounter instead of at the interpreter most of the time. |  |  |  |  |  |
| 2 | The trainee directly addressed the issues interpreted that were of concern to me. |  |  |  |  |  |
| 3 | The trainee acknowledges and responds to my beliefs, concerns, and expectations about my problems. |  |  |  |  |  |
| 4 | The trainee asked me questions in the first person (example: “Do you feel…” rather than “interpreter, can you ask him if he…”) |  |  |  |  |  |
| 5 | The trainee sat at a comfortable distance from me (not too close and not too far away.) |  |  |  |  |  |
| 6 | The trainee’s non-verbal body communications was reassuring (ie: mannerisms, facial expressions, and body language.) |  |  |  |  |  |
| 7 | Rate your overall satisfaction with the encounter. (Please circle only one number) | **5 4 3 2 1  Most Least** | | | | |
