# Supplementary material for: Partnering With Interpreter Services: Standardized Patient Cases to Improve Communication With Limited English Proficiency Patients
Source: MedEdPORTAL. 2019 May 20;15:10826. doi: 10.15766/mep_2374-8265.10826 (PMC6543860; doi:10.15766/mep_2374-8265.10826)
Supplement: Supplementary file 1 — A. Case 1 SP Information.docx B. Case 2 SP Information.docx C. Case 1 Resident Participant Information.docx D. Case 2 Resident Participant Information.docx E. Case 1 Physical Exam Sheet.docx F. Case 2 Physical Exam Sheet.docx G. UCI Interpreter Scale.docx H. UCI Interpreter Impact Rating Scale.docx I. Resident Session Evaluation Form.docx J. OSCE Workshop Schedule.docx K. UCI FORS Scale.docx L. Case 1 Observer Checklist.xlsx M. Case 2 Observer Checklist.xlsx [file mep-15-10826-s001.zip › I. Resident Session Evaluation Form.docx]

Appendix I – Resident Evaluation Form (Fall Version)

Dear Participant:

Please answer the following questions about your experience in today’s OSCE and your care of real patients:

1. How effective was today’s OSCE in mimicking a real patient encounter?

**1 (not effective) 2 3 4 5 (very effective)**

1. Rate your ability to get the information you needed from the patient in today’s OSCE scenario.

**1 (missing a lot of information) 2 3 4 5 (missing no information)**

1. When presented with a Spanish-speaking, Limited English Proficiency patient, I utilize a hospital interpreter (in person or via phone) for (check all that apply):
   1. The History and Physical (H&P)
   2. Daily rounds
   3. Status updates
   4. Brief/serial patient examinations throughout the day
   5. Family meetings
   6. Discharge talk
   7. Informed consent for procedures/blood products
   8. All of the above
   9. Other (please describe):
2. When I am pressed for time, I sometimes “get by” with my Spanish-speaking, Limited English Proficiency patients by doing the following (check all that apply):
   1. Utilizing a patient’s family member or friend who speaks English and is willing to interpret
   2. Using my Spanish language skills to the best of my ability
   3. Utilizing a web- or app-based translation service (e.g. Google translate)
   4. Asking a colleague (co-resident, nurse, BA, etc) who has better Spanish-language skills to interpret for me
   5. Other (please describe):

Appendix I – Resident Evaluation Form (Spring Version)

Dear Participant:

Please answer the following questions about your experience in today’s OSCE and your care of real patients:

1. How effective was today’s OSCE in mimicking a real patient encounter?

**1 (not effective) 2 3 4 5 (very effective)**

1. Rate your ability to get the information you needed from the patient in today’s OSCE scenario.

**1 (missing a lot of information) 2 3 4 5 (missing no information)**

1. Thinking back to your interactions with real patients in the fall of 2017, how would you rate your effectiveness in communicating with Spanish-speaking patients?

**1 (poor) 2 3 4 5 (outstanding)**

1. Thinking about your interactions with real patients in the past few weeks, how would you rate your effectiveness in communicating with Spanish-speaking patients?

**1 (poor) 2 3 4 5 (outstanding)**

1. How has your effectiveness with using an interpreter with Spanish-speaking patients changed over the past 6 months?

**1 (much worse) 2 3 4 5 (much improved)**

1. When presented with a Spanish-speaking, Limited English Proficiency patient, I utilize a hospital interpreter (in person or via phone) for (check all that apply):
   1. The History and Physical (H&P)
   2. Daily rounds
   3. Status updates
   4. Brief/serial patient examinations throughout the day
   5. Family meetings
   6. Discharge talk
   7. Informed consent for procedures/blood products
   8. All of the above
   9. Other (please describe):
2. When I am pressed for time, I sometimes “get by” with my Spanish-speaking, Limited English Proficiency patients by doing the following (check all that apply):
   1. Utilizing a patient’s family member or friend who speaks English and is willing to interpret
   2. Using my Spanish language skills to the best of my ability
   3. Utilizing a web- or app-based translation service (e.g. Google translate)
   4. Asking a colleague (co-resident, nurse, BA, etc) who has better Spanish-language skills to interpret for me
   5. Other (please describe):
