# Supplementary material for: Partnering With Interpreter Services: Standardized Patient Cases to Improve Communication With Limited English Proficiency Patients
Source: MedEdPORTAL. 2019 May 20;15:10826. doi: 10.15766/mep_2374-8265.10826 (PMC6543860; doi:10.15766/mep_2374-8265.10826)
Supplement: Supplementary file 1 — A. Case 1 SP Information.docx B. Case 2 SP Information.docx C. Case 1 Resident Participant Information.docx D. Case 2 Resident Participant Information.docx E. Case 1 Physical Exam Sheet.docx F. Case 2 Physical Exam Sheet.docx G. UCI Interpreter Scale.docx H. UCI Interpreter Impact Rating Scale.docx I. Resident Session Evaluation Form.docx J. OSCE Workshop Schedule.docx K. UCI FORS Scale.docx L. Case 1 Observer Checklist.xlsx M. Case 2 Observer Checklist.xlsx [file mep-15-10826-s001.zip › J. OSCE Workshop Schedule.docx]

**Interpreter OSCE Workshop Schedule**

|  | | |  |  |
| --- | --- | --- | --- | --- |
|  |  |  |  |  |
|  | Case 1 (Actor 1, Interpreter 1) | Case 1 (Actor 2, Interpreter 2) | Case 2 (Actor 3, Interpreter 3) | Case 2 (Actor 4, Interpreter 4) |
| GROUP 1 |  |  |  |  |
| 6:30-6:55 | Resident A | Resident B | Resident C | Resident D |
|  |  |  |  |  |
| GROUP 2 |  |  |  |  |
| 7:00-7:25 | Resident E | Resident F | Resident G | Resident H |
|  |  |  |  |  |
| GROUP 3 |  |  |  |  |
| 7:30-7:55 | Resident I | Resident J | Resident K | Resident L |
|  |  |  |  |  |
