# Supplementary material for: Partnering With Interpreter Services: Standardized Patient Cases to Improve Communication With Limited English Proficiency Patients
Source: MedEdPORTAL. 2019 May 20;15:10826. doi: 10.15766/mep_2374-8265.10826 (PMC6543860; doi:10.15766/mep_2374-8265.10826)
Supplement: Supplementary file 1 — A. Case 1 SP Information.docx B. Case 2 SP Information.docx C. Case 1 Resident Participant Information.docx D. Case 2 Resident Participant Information.docx E. Case 1 Physical Exam Sheet.docx F. Case 2 Physical Exam Sheet.docx G. UCI Interpreter Scale.docx H. UCI Interpreter Impact Rating Scale.docx I. Resident Session Evaluation Form.docx J. OSCE Workshop Schedule.docx K. UCI FORS Scale.docx L. Case 1 Observer Checklist.xlsx M. Case 2 Observer Checklist.xlsx [file mep-15-10826-s001.zip › K. UCI FORS scale.docx]

UCI School of Medicine
Faculty Observer Rating Scale (FORS)

Checklist for **Faculty** to complete on trainee after each encounter

Trainee’s Name: __________________ Faculty Name: ________________________

**Please rate the TRAINEE’S PERFORMANCE on each item using a scale of “1” (performed poorly or not at all) through “5” (outstanding performance throughout the encounter).**

|  |  | **Poor 🡪 Outstanding** |
| --- | --- | --- |
| 1 | The trainee adequately explained the purpose of the interview. | 1 2 3 4 5 |
| 2 | The trainee explained the interpreter’s role to the patient at the beginning. | 1 2 3 4 5 |
| 3 | The trainee asked the patient one question at a time. | 1 2 3 4 5 |
| 4 | The trainee listened to the patient without unnecessary interruption. | 1 2 3 4 5 |
| 5 | The trainee asked questions to clarify his/her own understanding of the patient’s answers. | 1 2 3 4 5 |
| 6 | The trainee presented information at a pace that was easy to follow for both patient and interpreter; that is, information was given in “digestible chunks.” | 1 2 3 4 5 |
| 7 | The trainee maintained direct eye contact with the patient (instead of the interpreter). | 1 2 3 4 5 |
| 8 | The trainee addressed the patient in the first person and not as “he/she.” | 1 2 3 4 5 |
| 9 | The trainee appropriately closed the encounter: at a minimum, asked the patient if he/she had any questions. | 1 2 3 4 5 |
| 10 | To what extent did the trainee keep the interpreter on track within his/her assigned role? | 1 2 3 4 5 |
| 11 | Global rating of trainee’s effectiveness in using the interpreter for the patient encounter. | 1 2 3 4 5 |
